# Supplementary material for: Time-series transcriptome analysis identified differentially expressed genes in broiler chicken infected with mixed Eimeria species
Source: Front Genet. 2022 Aug 8;13:886781. doi: 10.3389/fgene.2022.886781 (PMC9393255; doi:10.3389/fgene.2022.886781)
Supplement: Supplementary file 2 [file DataSheet1.ZIP › 4dpi_GO.Gsea.1625071243202/heat_map_corr_plot.html]

Heat map and correlation plot for TMM\_4dpi\_gct\_format\_4dpi\_gct\_format.Class\_4dpi.cls#PC\_versus\_NC  

Fig 1: heat\_map      
 Heat Map of the top 50 features for each phenotype in TMM\_4dpi\_gct\_format\_4dpi\_gct\_format.Class\_4dpi.cls#PC\_versus\_NC

  
  

Fig 2: Ranked Gene List Correlation Profile      
 Ranked list correlations for TMM\_4dpi\_gct\_format\_4dpi\_gct\_format.Class\_4dpi.cls#PC\_versus\_NC

  
  
    
